# Looking at the big picture: understanding how the oviduct’s dialogue with gametes and the embryo shapes reproductive success

**DOI:** 10.21451/1984-3143-AR2018-0036

**Published:** 2018-08-03

**Authors:** Beatriz Fernandez-Fuertes, Beatriz Rodríguez-Alonso, José María Sánchez, Constantine A. Simintiras, Patrick Lonergan, Dimitrios Rizos

**Affiliations:** 1 School of Agriculture and Food Science, University College Dublin, Belfield, Ireland; 2 Departamento de Reproducción Animal, Instituto Nacional de Investigaciones y Tecnologia Agrarias y Alimentarias, Madrid, Spain

**Keywords:** cattle, embryo, gametes, interaction, oviduct.

## Abstract

The oviduct is a tubular organ comprising three distinct anatomical regions (the infundibulum, the ampulla and the isthmus) connecting the ovary and the uterus. Oviductal function is regulated by ovarian hormones, gametes, and embryo-derived factors, for optimally facilitating key reproductive events. A cross- talk is established between the oviduct and the gametes and embryo and this dialogue shapes the microenvironment in which gamete transport, fertilization, and early embryonic development occur. This review aims to address each participant in this conversation in a holistic manner by delineating several advances in the field within the greater context of understanding how oviduct-gamete and oviduct-embryo dialogue shape reproductive success and furthermore how this knowledge can be applied *in vitro*.

## Introduction

Successful blastocyst production following *in vitro* fertilization (IVF) and embryo culture (Gordon, 2003), coupled with the fact that pregnancies can be achieved after IVF embryo transfer to non-mated synchronized recipients (Lonergan *et al*., 2016), indicate that oviduct exposure is not essential for early embryo development. Thus, this has led to the view that the oviduct is a passive conduit for gametes and the early embryo(s). However, a significant body of evidence demonstrates that the oviduct is a dynamic organ. The luminal microenvironment is influenced by ovarian hormones, gametes, and embryo-derived factors, for the purpose of *optimally* facilitating key reproductive events - to the extent that a pathophysiological peri-conceptual milieu can result in embryo loss, or even adverse lifelong effects (Fazeli, 2008).

Following ovulation and/or insemination, a cross-talk is established between the oviduct and the gametes and embryo. This dialogue will shape the microenvironment in which gamete transport, fertilization, and, if successful fertilization takes place, early embryonic development occur. Studying each conversation participant in isolation facilitates research; however, to fully understand complex oviduct dynamics, a more holistic view is necessary. This review aims to achieve this by delineating several advances in the field within the greater context of understanding how oviduct-gamete and oviduct-embryo dialogue shape reproductive success.

## Oviduct anatomy and physiology: epithelial and fluid cyclic changes

The oviduct is a tubular organ comprising three distinct anatomical regions: 1) the infundibulum, 2) the ampulla, and 3) the isthmus, adjoining the uterus at the utero-tubal junction (UTJ) - all with different, yet equally critical, roles.

The oviduct epithelium comprises secretory (most abundant in the isthmus) and ciliated cells (most abundant in the infundibulum and ampulla; Yániz *et al*., 2000). The cause of the regional variation in cell type distribution is unknown; however, both lineages derive from embryonically-labelled PAX8+ (a secretory cell marker) cells (Ghosh *et al*., 2017). Therefore, secretory cells are the oviduct epithelium progenitors, with the potential to self-renew or differentiate into ciliated cells upon 17β-oestradiol (E2) stimulation (Comer *et al*., 1998).

The ampullar and the infundibular epithelium undergoes cycle-dependent changes - surface fold amplitude, cell populations, gene expression, and overall physiology vary in response to ovarian steroid fluctuations (Yániz *et al*., 2000; Cerny *et al*., 2015). Interestingly, the isthmic epithelium undergoes few changes throughout the cycle (Yániz *et al*., 2000), despite estrogen receptor alpha (ERα) and progesterone receptors A and B (PRA and PRB) being more abundant in the isthmus than the ampulla and infundibulum (Okada *et al.,* 2003). This suggests that the region- specific differences are not only due to differences in epithelial gene expression, but also likely due to the level of exposure to locally secreted factors - specifically from the ovary, ovulated follicle and consequent corpus luteum, via the ovarian artery and the oviductal ostium. This counter-current transfer is thought to underpin oviduct endocrine regulation (Hunter, 2012), and could explain why the ipsilateral oviduct contains higher concentrations of P4 during the luteal phase, relative to its contralateral counterpart (Wijayagunawardane *et al*., 1998; Lamy *et al*., 2016). It is important to note, however, that there is conflicting evidence regarding differences in abundance of other hormones such as E2, between ipsilateral and contralateral regions (Wijayagunawardane *et al*., 1998; Lamy *et al*., 2016). This local delivery system may act to coordinate oviductal tissue changes in step with the pre-ovulatory maturation of the oocyte within the Graafian follicle, and the capacitation of sperm. During the follicular phase, infundibular and ampullar folds reach maximum amplitude (greatest surface area to volume ratio) and exhibit numerous ciliated cells in the apical areas (Yániz *et al*., 2000), with secretory cells clustered basally, between folds. At this stage, genes involved in cell cycle, cholesterol biosynthesis, cell division, mitosis, and protein folding - responsible for proliferation and secretory activity - are upregulated (Cerny *et al.*, 2015). High E2, characteristic of the preovulatory phase, is thought to be responsible for proliferative epithelial activity (Steffl *et al*., 2008). Indeed, mitotic activity in the isthmus and ampulla is highest during the follicular phase and around ovulation (Ito *et al*., 2016). In addition, high E2-low P4 treatment induces morphological changes and increased P4 receptor (PR), estrogen receptor 1 (ESR1), oviductal glycoprotein 1 (OVGP1), and heat shock protein ember 90kDa member 1 (HSP90B1) gene expression in porcine oviduct cells (Chen *et al*., 2013). However, *in vitro* studies reported no increase in proliferation after FSH, LH (in baboon and mouse), or E2 treatment (in the baboon, mouse, and pig; King *et al.,* 2011; Chen *et al*., 2013), suggesting that additional factors may participate in epithelial remodelling.

At ovulation, expelled follicular fluid contacts the ipsilateral oviduct (Hansen *et al*., 1991), inducing increased ciliary beat frequency (CBF), therein aiding oocyte transit to the site of fertilization (Lyons *et al*., 2006). Ovulation, furthermore, induces double-strand DNA breaks in the oviduct epithelial cells (OEC), and increases epithelial macrophage infiltration (King *et al*., 2011). Interestingly, these macrophages associate with oviduct epithelia adjacent to the cumulus-oocyte complex (COC), which may be important as they secrete cytokines that could interact with the oocyte and the early embryo (Schäfer-Somi, 2003).

After ovulation, the P4 rise associated with the luteal stage, results in decreased oviduct mucosal fold amplitude, with secretory cells beginning to dominate the luminal landscape (Abe and Oikawa, 1993; Yániz *et al*., 2000). Epithelial exposure to elevated P4 leads to cell atrophy, decreased cell height, secretory granule loss, and cell death (Steffl *et al.*, 2008). The oviduct epithelium during the luteal phase is also characterised by a downregulation of genes involved in cell communication, blood vessel development, innate and humoral immune responses, complement activation, and an upregulation of genes involved in focal adhesion formation, cell growth regulation, and fatty acid metabolism, amongst others (Hess *et al*., 2013). These changes are indicative of an environment required to support semi-allogeneic embryo development.

## Oviduct fluid dynamics

Oviduct fluid (OF) formation is a spatio- temporally dynamic process. The spatial secretory profile is influenced by 1) the secretory cell proportion, which increases longitudinally from infundibulum to isthmus (Leese, 1983), and 2) the secretory mucosal surface area, which decreases as the oviduct tapers toward the UTJ. The most pronounced secretory portion of the oviduct is subject to debate. Whilst secretory cells dominate the isthmic luminal landscape (~70%), in contrast to ~50% in the ampulla (Crow *et al*., 1994), primary metabolites have been detected in the ampulla at 1.8 times their isthmic concentration - presumably owing to the relative secretory mucosal surface area of the ampulla being ~1.8 times greater (Leese, 1988). Factoring both surface area and secretory cell population, however, the ampulla has a secretory index of 0.9 (0.5 x 1.8) compared to the isthmic 0.7 (0.7 x 1.0) (adapted from Abe, 1996). This is physiologically counter-intuitive, given that the embryo migrates through the isthmus following fertilisation at the ampullary-isthmic junction.

In addition to spatial variability, OF composition and volume vary temporally as a function of the oestrous cycle, mediated by steroid hormones acting on the oviduct both directly and indirectly (Aguilar and Reyley, 2005). This was functionally demonstrated first by Bishop (1956) who ligated anaesthetised rabbit oviducts at the UTJ and vertically cannulated the ostium, measuring pressure as a function of fluid formed. At oestrous, oviducts produced 0.79 ml over 24 h, whereas ovariectomised subjects secreted 0.14 ml over the same period. Importantly, secretion rates were restored in ovariectomised rabbits following exogenous E2 supplementation, and secretion volume and pressure declined during pregnancy. Hugentobler *et al*. (2008) performed a similar study in heifers by catheterising the exteriorised oviduct during surgery. Whilst secretion rates declined from day 0 (1.9 ± 0.3 µl/min; n = 7 ± SEM) to day 6 (1.2 ± 0.3 µl/min; n = 7 ± SEM), differences were non-significant.

The primary OF formation mechanism is osmotic water transfer secondary to solute transit, the dominant of which is Cl^-^ (Dickens *et al*., 1993; Leese *et al*., 2001). K^+^ flux is also likely important for moving water apically (Dickens and Leese, 1994). The fact that oviduct epithelia exhibit an inherently relatively low transepithelial resistance (Leese and Gray, 1985) is indicative that paracellular fluid transport also contributes to OF formation and composition (Simintiras and Sturmey, 2017). It is also worth noting that, under physiological conditions, OF composition is influenced by peritoneal and follicular fluid entry from the abdominal cavity, and uterine fluid (UF; Leese, 1988.

## OF composition Ions

OF ionic composition is highly conserved across mammals, with K+ consistently elevated relative to plasma levels (Aguilar and Reyley, 2005). In cattle, K+ is highest in OF at oestrus (Olds and VanDemark, 1957), and in mice, more pregnancies were established by IVF when using a culture medium high in K+ (Quinn *et al*., 1985) - the basis of synthetic oviduct fluid (SOF), now also used for cattle embryo production (Gandhi *et al*., 2000). Ca2+ is also highest in bovine OF around ovulation and is interestingly more abundant in the isthmus than the ampulla (Grippo *et al*., 1992). This longitudinal variation is unlike Mg2+ which does not appear to vary spatially but rather temporally (Grippo *et al.,* 1992). It is also interesting to note that the ionic composition and rate of secretion of bovine OF differs considerably to that of uterine fluid (Hugentobler *et al*., 2007).

## Protein

The OF protein source is twofold: 1) basal vasculature ‘filtration’ and 2) epithelial synthesis and secretion (Aguilar and Reyley 2005). OF protein levels are ~10 - 15% of that of plasma (Leese, 1988), with serum albumin and serum immunoglobulin G comprising approximately 95% of this total (Oliphant *et al*., 1978). Other proteins identified include high-density lipoproteins, secreted during the follicular phase, and presumed to bind sperm membrane cholesterol as part of the capacitation process (Ehrenwald *et al.,* 1990).

Further to spatially-regulated protein secretions, a temporal pattern of protein secretion is evident (Nieder and Macon, 1987; Abe, 1996), as discussed below in the context of the best studied and characterised protein of the oviduct: OVGP1, reported as the major secretory glycoprotein which is synthesized and secreted exclusively by the oviduct (Buhi, 2002). OVGP1 is consistently observed in the ampulla across species and enters the lumen via epithelial secretory granule exocytosis (Avilés *et al*., 2010). OVGP1 has also been identified in *in vitro* derived bovine, porcine, and murine oviduct fluid (Chen *et al*., 2017; Simintiras *et al.*, 2017).

OVGP1 secretion *in vivo* is cycle-dependent and thus correlates with the aforementioned epithelial differentiation states (Verhage *et al*., 1988); however, OVGP1 production and secretion patterns differ between species. In the goat it is expressed in the infundibulum and ampulla during the follicular phase (Abe *et al*., 1995) - *i.e*. around the time of fertilisation but not at the site of fertilisation, whereas in the rat it is secreted predominantly in the isthmus, where the sperm reservoir is located (Abe, 1996). In the bovine, OVGP1 is found in the isthmus and ampulla, the respective sites of sperm capacitation and fertilisation (Lefebvre *et al*., 1997) during the follicular phase. Ovine OVGP1 is exclusively produced by the ampulla (Gandolfi *et al.,* 1991), in greatest amounts at oestrus (DeSouza and Murray, 1995).

Advances in proteomic methods (Simintiras and Forde, 2017) such as mass spectrometry, will undoubtedly lead to a clearer picture of the oviduct proteome, based on empirical data as opposed to gene expression extrapolations. For instance, a recent study by Acuña *et al*. (2017) found almost 5000 genes expressed in the porcine oviduct, of which only 7% corresponded to secretory proteins, and 11% to membrane proteins - *i.e.* products with the potential to directly influence the offspring.

## Extracellular vesicles

An additional new area of research lies in luminal extracellular vesicles (EVs). The term EV encompasses different vesicle types, released by somatic cells, that are present in body fluids, and contain bioactive molecules (i.e. mRNAs, small ncRNAs - such as miRNA, proteins, carbohydrates, and lipids; Raposo and Stoorvogel, 2013). EVs are important for intercellular communication, playing a key role in the regulation of physiological and pathological processes (Thery, 2011). EVs can horizontally transfer mRNAs to other cells, which can then be translated into functional proteins at the new location (Hergenreider *et al*., 2012). EVs have been identified *in vivo* in several body fluids including amniotic fluid, urine, and blood (Simpson *et al*., 2008). Until recently, the study of reproductive EVs in mammals was limited to follicular fluid (Silveira *et al*., 2012), uterine fluid (Ng *et al*., 2013; Burns *et al*., 2014), and seminal plasma (SP; Piehl *et al*., 2013). Burns *et al*. (2016) demonstrated that EVs emanate from both the conceptus trophectoderm and uterine epithelia, and are involved in intercellular communication between these tissues during pregnancy establishment in sheep. Recent studies from our group showed that EVs obtained from bovine OECs cultures *in vitro* (Lopera-Vásquez *et al*., 2016) and from bovine OF (Lopera-Vasquez *et al*., 2017) substantially improved *in vitro* produced blastocyst quality, measured in terms of cryotolerance, differentially cell count and mRNA abundance of specific genes. However, it was evidenced that EVs obtained from *in vivo* and *in vitro* bovine OECs differ in their protein content, with some proteins known to be involved in reproductive function differently abundant in EVs from *in vivo* compared to *in vitro* origin (Almiñana *et al*., 2017). Thus, oviductal EVs from different origins may differ in their ability to mediate key processes such as sperm-oocyte binding and fertilization; for greater detail see (Pérez-Cerezales *et al*., 2018).

## Oviduct-gamete interactions

The response of the oviduct to sperm or oocytes differs, but both the male and female gamete induce changes in the oviductal proteome (Georgiou *et al*., 2005). Oviduct-gamete communication is an intricate dialogue leading to the fine regulation of sequential processes resulting in successful fertilization. The main oviduct-driven events in gamete physiology are detailed below.

## Oocyte transportation to the site of fertilization

At ovulation, the COC is expelled into the peritoneal cavity and guided through the infundibulum into the ampulla of the oviduct. Once contact is established between the COC and the oviduct epithelium, ciliated cells transport the COC to the ampulla. In addition to the OF current created by ciliary beating, COC adhesion to ciliary cells is essential for gamete transport (Lam *et al*., 2000). Adhesion is mediated by the cumulus cells, as their removal prevents oocyte pick-up, due to the zona pellucida not interacting with the epithelia (Mahi-Brown and Yanagimachi, 1983). The granules and filaments of the cumulus extracellular matrix adhere to the glycocalyx of ciliary crowns at the infundibular ciliary tip (Lam *et al*., 2000). Ciliary beating weakens this adhesive interaction, such that the COC is never completely released, yet rolls into the ampulla. The importance of ciliary cells in this process is highlighted by the fact that women with Kartagener syndrome, a genetic disorder causing defects in global ciliary action, exhibit impaired fertility (Afzelius and Eliasson, 1983; McComb *et al.,* 1986).

Interestingly, mating induces changes in oviduct ER signalling, which is directly involved in oocyte transport acceleration (Orihuela *et al*., 2009). This could be a mechanism for ensuring that the oocyte and sperm meet at an appropriate time, and represents an example of how the sperm, oocyte, and oviduct interact to ensure successful fertilization.

## Oviduct sperm reservoir formation

In many mammalian species, sperm bind to the isthmic epithelium to establish a sperm reservoir. Different studies have linked the formation of this storage reservoir to the prevention of polyspermy, or maintenance of sperm motility and fertility until ovulation (Suarez, 2006). Indeed, sperm incubated with OECs are capable of developing hypermotility, and maintain their fertilising capacity for 30 h, in contrast to sperm incubated in isolation (Pollard *et al.*, 1991). In the bovine, sperm-oviduct interactions are mediated by fucose residues present throughout the oviduct during oestrus (Lefebvre *et al*., 1997). Only uncapacitated sperm can bind to the oviduct (Lefebvre and Suarez, 1996). In fact, Ca2+ influx and tyrosine phosphorylation in sperm are reduced or inhibited whilst bound, likely keeping them uncapacitated (Töpfer-Petersen *et al*., 2002). Reservoir release likely occurs via plasma membrane modification, leading to the loss of oviductal binding proteins, and hyperactivation of motility (Suarez, 2006). The signals that maintain sperm quiescence and that activate capacitation remain unknown; however, it is plausible that ovarian cues from the dominant or ovulated follicle stimulate the oviduct epithelium to secrete factors that regulate sperm physiology. This would explain why OF from oestrus cows is more successful in inducing sperm capacitation than fluid collected from other stages of the cycle (Parrish *et al.,* 1989).

Once sperm disengage from the reservoir they still have to make their way to the site of fertilisation. So far, four mechanisms have been proposed to guide sperm to the proximity of the oocyte, all of which are driven by the female environment: peristaltic pumping, thermotaxis, rheotaxis, and chemoattractant gradient (Suarez, 2006). The smooth muscle contractions of the oviduct, especially in the isthmus, not only propel sperm, but also create OF currents (Ishikawa *et al.*, 2016). Bull sperm have been shown to orientate their heads against a current when flow velocity reaches 15 µm/s (Tung *et al*., 2015). While the rate of fluid flow in the bovine oviduct is unknown, in mice it is 18 ± 1.6 µm/s (Miki and Clapham, 2013). In addition to OF flow, thermotaxis has been proposed as a long-range guiding mechanism. In pigs and rabbits, a temperature drop in the isthmus is observed at ovulation (Hunter and Nichol, 1986; Bahat *et al*., 2005). Capacitated sperm seem able to sense temperature differences and orientate their swimming towards warmer temperatures (Bahat *et al*., 2012; Pérez-Cerezales *et al.*, 2015a), leading them to the site of fertilisation. The final guidance system, chemotaxis, is likely limited to short distances, within the order of millimetres (Pérez-Cerezales *et al*., 2015b). Many substances have been proposed as sperm chemoattractants (reviewed by Eisenbach and Giojalas, 2006); however, due to multiple technical difficulties in chemotactic studies, the data are inconclusive.

## Sperm capacitation and hyperactivation

The fertilising ability of sperm is suppressed until capacitation, a process comprising physiological changes, which physiologically occurs in the female reproductive tract (Yanagimachi, 1994). These include: flagellar motility hyperactivation, regulation of signal transduction pathways enabling chemoattractant responsiveness and acrosome-oocyte reactivity (Florman and Fisore, 2014).

Capacitation seems to be initiated by cholesterol efflux (Visconti *et al*., 2002). Cholesterol removal requires extracellular bicarbonate and cholesterol acceptors, such as albumin, one of the major OF proteins (Flesch *et al*., 2001). Cholesterol extraction increases membrane fluidity and ion permeability (Flesch and Gadella, 2000; Khorasani *et al*., 2000, and initiates diffusion, and possibly formation, of acrosomal lipid raft-like structures containing ZP-binding molecules (Khalil *et al*., 2006).

In addition to membrane architecture changes, the oviduct can alter sperm motility patterns which can be recapitulated *in vitro*. Hyperactivated motility, seen in most sperm recovered from the ampulla, requires elevated Ca2+ (Colás *et al*., 2010) and enables sperm to penetrate OF, the cumulus intercellular matrix, and the ZP. Plasma membrane Ca2+-ATPase 4a (PMCA4a), the major Ca2+ efflux pump in murine sperm, is present in OF EVs (Al-Dossary *et al*., 2013), and plays an important role in sperm motility, as its absence leads to an inability to hyperactivate (Okunade *et al*., 2004). EV PMCA4a is enzymatically active and can be transferred to sperm, as evidenced by increased activity following EV interaction (Bathala *et al*., 2018). On the other hand, CatSper (cation channel of sperm is the major Ca2+ entry pathway controlling sperm hyperactivation in different mammalian species (Ren *et al*., 2001; Quill *et al*., 2003; Johnson *et al*., 2017). Nanomolar concentrations of P4, diluted ZP preparations, or bovine serum albumin (BSA) can activate CatSper, inducing increased intracellular Ca2+ (Xia and Ren, 2009a, b; Lishko *et al.,* 2011; Smith *et al*., 2013). Therefore, the emerging theory is that CatSper is essential for sperm hyperactivation and is controlled by oviduct signals, depending on sperm location and phase of the cycle (Kirichok and Lishko, 2011; Johnson *et al*., 2017).

## Fertilization

Soon after the oocyte and sperm meet in the ampulla, fertilization occurs - a complex process requiring an intimate association between the gametes, such that the sperm can penetrate the ZP and plasma membrane, and deliver the paternal DNA. Although some of the key players of these interactions remain unknown, several OF-derived factors are thought to be involved. Perhaps one of the most studied is OVGP1. As mentioned above, OVGP1 has been identified in the OF of numerous mammals, and has been shown to bind to the ZP (O’Day-Bowman *et al*., 1996; Coy *et al*., 2008). Interestingly, the role of this protein appears to differ between species. Porcine and bovine oocyte incubation with OF leads to decreased sperm bound to the ZP (Coy *et al*., 2008). Moreover, in the same species, OF-derived OVGP1 and heparin-like glycosaminoglycans seem to increase ZP resistance to enzymatic digestion and sperm penetration, contributing to the control of polyspermy (Coy *et al*., 2008; Algarra *et al*., 2016). OVGP1 can also bind to sperm to mediate changes involved in the process of capacitation and acrosome reaction (Choudhary *et al*., 2017); another example of how the oviduct can synchronise the capacitation status of the sperm to ensure that fertilization occurs under optimal circumstances.

## Immune response modulation

The immune system of the reproductive tract is uniquely required to protect the mother against pathogens, whilst allowing symbiosis with allogeneic sperm and the semi-allogeneic embryo and fetus. The mechanisms regulating immunological tolerance towards paternal antigens and the embryo have not been completely elucidated. However, the oviduct epithelium seems to play an important role. Sperm incubation with OEC-conditioned media decreases their phagocytosis by neutrophils *in vitro* (Marey *et al*., 2014). Prostaglandin E2 (PGE2), alpha-1 acid glycoprotein (AGP), (BSA), and the combination of AGP or BSA with other OF components are predicted to regulate this decreased phagocytosis (Kowsar *et al*., 2017). It seems that live sperm are involved in the regulation of this protective response, as sperm binding to OECs induces them to produce PGE2, and the anti-inflammatory cytokines TGFB1 and IL10 (Yousef *et al.,* 2016). In contrast, dead or abnormal sperm fail to induce PGE2 secretion (Kodithuwakku *et al*., 2007).

The role of SP in modulating reproductive immune responses has been gaining interest lately. The absence of SP at insemination in mice leads to decreased embryo development in the oviduct, embryo implantation, and placental development (Bromfield *et al*., 2014). The positive effect of SP is thought to be attributable to its immunoregulatory properties - it induces antigen specific Treg cell expansion, as well as tolerogenic dendritic cell expansion, considered important in immune tolerance to paternal antigens in the embryo (Robertson *et al*., 2009; Guerin *et al*., 2011; Shima *et al*., 2015). In addition, granulocyte- macrophage colony-stimulating factor (CSF2), leukemia inhibitory factor (LIF), interleukin 6 (IL6), and tumor necrosis factor-related apoptosis-inducing ligand (TRAIL), embryokines important to embryo quality, can be regulated by SP exposure in the oviduct (Bromfield, 2016). Thus, seminal plasma may help shape an optimal environment for the early embryo. However, evidence for a significant role for seminal plasma in pregnancy establishment in cattle is not clear. To date, the only study that has looked at the effect of SP or transforming growth factor beta (TGFβ) (thought to be responsible for the beneficial effects of SP in rodents) in cattle pregnancy outcome, concluded that this factor (but not SP as a whole) had a positive effect only when reproduction was suboptimal (Odhiambo *et al*., 2009).

## Oviduct-embryo communication

Following fertilization, the bovine zygote spends ~4 days in the oviduct until migrating to the uterus as a 16-cell stage embryo (Hunter, 2012). During this period, the oviduct provides a nourishing environment conducive to embryo development comprising simple and complex carbohydrates, ions, lipids, phospholipids and proteins (Avilés *et al*., 2010). In addition, the oviduct is also responsible of transporting the embryo to the uterus through muscular and ciliary activity.

**Figure 1:**
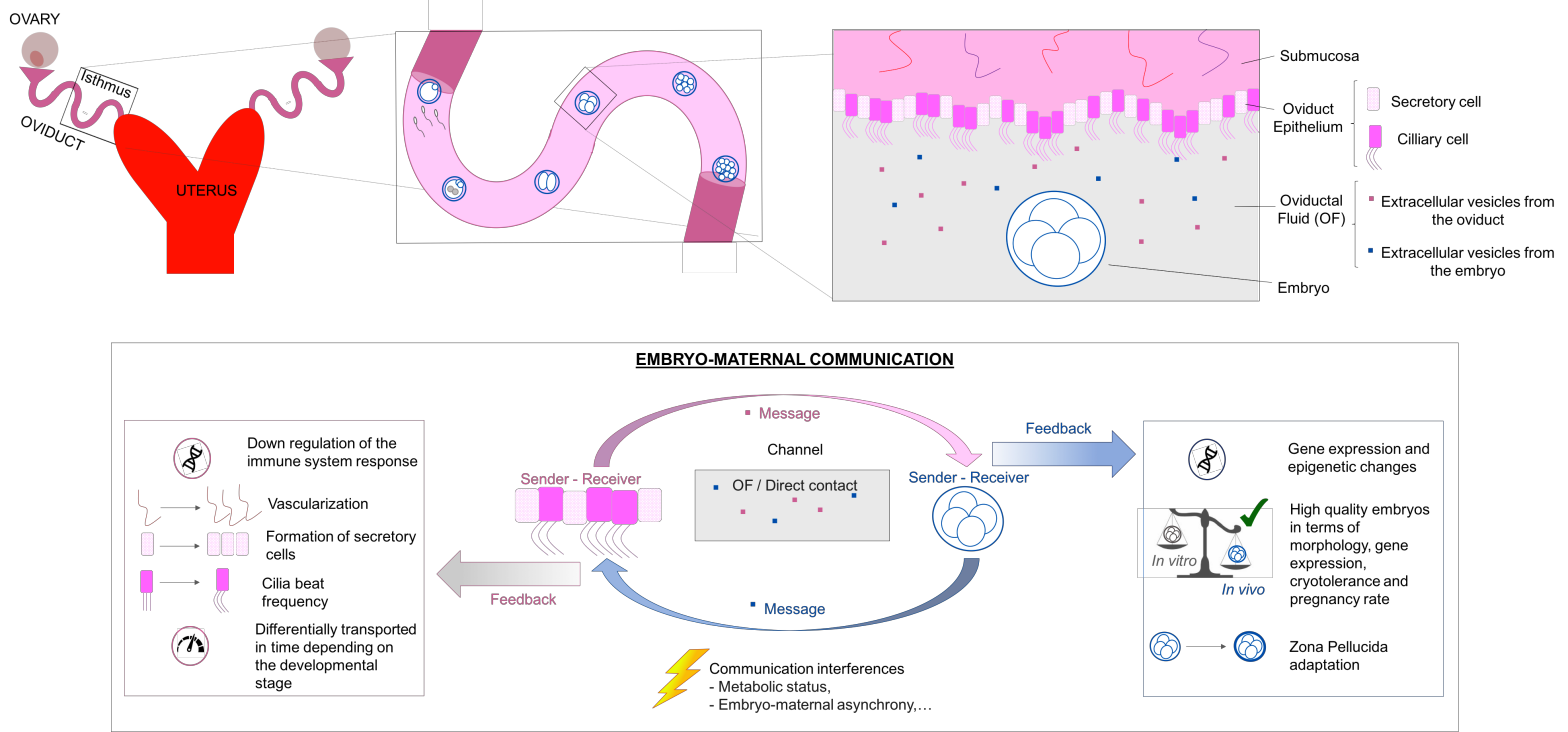
Schematic representation of embryo-maternal communication in the oviduct.

Whilst uterine-embryo dialogue has been extensively studied, relatively little is still known about oviduct-embryo communication. Our current understanding is that this phenomenon is a two-way process (Fig. 1), *i.e.* signals can be sent and received from both the oviduct and the embryo; however, these remain largely undefined. Our group has recently described bone morphogenetic proteins (BMPs) as participants in a signalling pathway involved in oviduct- embryo cross-talk *in vitro* (García *et al*., 2017). Embryo- oviduct interaction *in vitro* induces transcriptional changes of BMP signalling components, both through direct and indirect contact (Hamdi *et al*., 2018), indicating that the signal is released in OF. Thus, analysing early embryo-maternal interactions involves studying OF in addition to the embryo, oviduct epithelium, and the direction of the communication.

In vivo *modelling*

Although tremendous advances have led to improved *in vitro* models for studying embryo-oviduct interactions, such models remain limited in their ability to fully mimic *in vivo* conditions (Lonergan and Fair, 2008). Therefore, animal models are crucial to holistically understanding the physiology and pathology surrounding early embryo development.

In our laboratory, we have investigated the effect of different embryo culture environments (*in vitro, in vivo* in surrogate ovine oviducts, and *ex vivo* in the murine oviduct) on blastocyst development and quality, finding that culture in the oviduct (*in vivo* and *ex vivo*) improves embryo quality in terms of morphology, gene expression, and cryotolerance (Enright *et al*., 2000; Rizos *et al*., 2002; Lonergan *et al*., 2003). Interestingly, heterologous embryo culture can support early embryo development, resulting in the establishment and maintenance of pregnancy, although only the ovine oviduct has been routinely used for bovine embryo culture (Lazzari *et al*., 2010). A new approach for culturing *in vitro* and/or *in vivo* produced embryos in the homologous bovine oviduct *in vivo* by transvaginal endoscopy has been introduced successfully (Besenfelder *et al*., 2010). Using this technique, Wetscher *et al*. (2005) and Havlicek *et al*. (2010), found that short and long-term *in vivo* culture of *in vitro* produced embryos in the homologous bovine oviduct enhanced blastocyst quality, in terms of cryotolerance, relative to blastocysts grown entirely *in vitro*. Moreover, total blastocyst yields were similar to previous data derived using surrogated heterologous oviducts as a temporary incubator (Enright *et al*., 2000; Rizos *et al*., 2002; Lonergan *et al*., 2003; Lazzari *et al*., 2010).

A key milestone in early embryo development is embryonic genome activation (EGA). Using alternative *in vivo* and *in vitro* culture conditions for blastocyst production, Gad *et al*. (2012) demonstrated that *in vitro* conditions at the time of major EGA critically influence the transcriptome of the subsequent blastocysts. Furthermore, the methylation pattern of *in vitro* derived blastocysts differs from *in vivo* counterparts (Gad *et al*., 2012). This was demonstrated in the bovine by Salilew-Wondim *et al*. (2015), who transferred *in vitro* zygotes, 4-cell, and 16-cell embryos into recipient heifer oviducts. Resulting blastocysts were recovered on day 7 and compared with blastocysts produced *in vitro*. The degree of DNA methylation dysregulation in the promoter and/or gene body regions was correlated positively with *in vitro* culture duration.

Due to the early embryo being usually described as somewhat autonomous up to the blastocyst stage (*i.e*. does not need contact with the maternal reproductive tract), one could argue about the influence of maternal-embryonic asynchrony on embryo development. To investigate this further, our group endoscopically transferred day 1 *in vitro* produced bovine zygotes to the oviducts of heifers either synchronous with the embryos (at day 1 post-ovulation), or asynchronous (at day 3 post-ovulation), prior to embryo recovery on day 4 (8- to 16-cell stage), day 7 (morula-blastocyst), and day 15 (elongated conceptus). Interestingly, asynchrony had a negative impact on early embryo survival and development (Rodríguez-Alonso *et al*., 2018a), unlike in the uterus, wherein the transfer of a blastocyst to an advanced uterus results in accelerated embryo development (Randi *et al*., 2016).

Another study from our group assessed the contribution of the oviduct to poor fertility in postpartum dairy cows - a disorder linked with suboptimal follicle development, oocyte quality, sperm transport and fertilization, reproductive tract environment, and/or a combination of these (Lonergan *et al*., 2016) - and found significantly lower blastocyst yields when *in vitro* produced zygotes were transferred to the oviducts of lactating (~ day 60 postpartum *vs*. dry cows (Maillo *et al*., 2012) and heifers (Rizos *et al*., 2010).

Today, most of the studies related to the maternal-embryo interactions in the oviduct reflect the effect on the embryo, meanwhile there are only few reported the converse effect on the oviduct (reviewed by Maillo *et al*., 2016). Even more, most of them have been performed in poly-ovulatory species - *e.g.* murine and porcine - in which the presence of multiple embryos presumably magnifies the signal for altering OEC gene expression (Chang *et al.,* 2000; Lee *et al.*, 2002; Almiñana *et al.,* 2012). In an effort to dissect the directionality of oviduct-embryo dialogue in the mono- ovulatory species, Smits *et al*. (2016) reported a local influence of a single embryo on the transcriptome of the equine oviduct epithelium; while in bovine Maillo *et al*., (2015) was unable to detect differences in the oviduct isthmus transcriptome in the presence of a single embryo. However, when up to 50 embryos were endoscopically transferred into heifer oviducts, with the aim of amplifying embryo-derived signals, OEC transcriptomic differences became apparent, mostly related to the immune system response. Thus, the physiological local embryo-oviduct interaction may be undetectable using current technologies owing to the relatively small and localised response elicited.

To tease this out, we recently isolated ipsilateral oviducts from single-ovulated artificially inseminated heifers post-mortem on day 2.5 post-estrus. These were subsequently sectioned (into 2 cm lengths) and flushed for embryo retrieval (2-cell). The expression of 10 genes previously shown to be differentially expressed between the isthmus of pregnant and cyclic heifers (Maillo *et al*., 2015), was assessed. Differences were found both where the embryo was located and proximally, *i.e.* where the embryo had passed (Rodríguez-Alonso *et al*., 2018b).

## In vitro modelling

Owing to technical limitations surrounding OF sampling *in vivo* (see Leese *et al*., 2008) coupled with logistical issues, and the high costs associated with *in vivo* studies, *in vitro* models are pivotal to studying oviduct physiology. *In vitro* modelling furthermore enables investigations of greater environmental manipulation, (Ulbrich *et al*., 2010). OECs are currently generally cultured *in vitro* as basic monolayers or cell suspensions (Lopera-Vásquez *et al.* 2016), polarized two-dimensional monolayers (Chen *et al*., 2017; Jordaens *et al*., 2017; Simintiras *et al*., 2017), or three- dimensional monolayers (Ferraz *et al*., 2017a, b).

Despite *in vitro* OEC de-differentiation and morphological characteristic loss (Rottmayer, *et al*. 2006), including height reduction, cilia and secretory granule loss, and bulbous protrusions (Thibodeaux, *et al.* 1992, Walter 1995), *in vitro* modelling presents an opportunity to detect essential and functional candidate genes in embryo-maternal dialogue (Schmaltz-Panneau *et al.* 2014) that are difficult to study *in vivo*, and the capacity to investigate OF formation and regulation free from systemic effects (Simintiras *et al*., 2017). The latter, coupled with OEC-conditioned media (Ramos- Ibeas *et al.* 2014), offer scope for improving *in vitro* embryo culture, particularly as co-culture is associated with a lack of reproducibility, biosanitary risk (Guerin *et al.* 1997), do not contain foreign cells, and contain embryotrophic factors (Ramos-Ibeas *et al.*, 2014). We recently reported that conditioned media from extended bovine OEC monolayer cultures had a consistently positive effect on blastocyst quality when used during IVC (Lopera-Vásquez *et al.* 2016).

One limitation of *in vitro* work is an inherent behavioural variability between cell populations; however, a promising solution is the use of immortalised cell lines that maintain many primary culture attributes (Ulbrich *et al.* 2010). Another development is the short-term (24 h) epithelial cell suspension culture, in which OECs maintain morphological characteristics as well as gene markers present *in vivo* such as OVGP1, E2 and P4 receptors (Rottmayer, *et al.* 2006). However, suspended cells do not adhere and mitosis does not occur (Walter, 1995).

The OEC polarized system consists of culturing the cells on inserts to allow media access from both basolateral (vasculature mimic) and apical (luminal mimic) sides, therein also maintaining the natural asymmetrical nature of the epithelium. This system preserves detailed morphological features of the porcine oviduct and oviduct-specific markers (Miessen *et al*., 2011). Bovine OECs cultured in this way have been used to model elevated non-esterified fatty acid metabolic stress (Jordaens *et al*., 2015, 2017) in addition to testing the barrier properties of the oviduct epithelium to dietary-derived embryotoxins (Simintiras and Sturmey, 2017). Another category of such polarized culture is the air-liquid interface (ALI) system in which medium is exclusively supplied basolaterally, allowing the formation of oviduct fluid surrogate or *in vitro* derived oviduct fluid (Simintiras *et al*., 2017), in the apical chamber. Epithelia derived from human, porcine, and bovine oviducts maintain polarity and an *in vivo*- like morphology when cultured like this long-term (Chen *et al*., 2013, 2017; Levanon *et al*., 2010).

Moreover, Chen *et al*. (2017) reported that ALI supports development *in vitro* in the OFC, of porcine, murine, and bovine embryos. However, blastocyst rates were inferior to current optimized standard IVP procedures, suggesting a need for further model improvement by simulating physiological hormonal changes, and developing a sequential culture system using oviduct as well as uterine epithelial cells (Chen *et al*., 2017).

Very recently, the use of three-dimensional (3D) printing in combination with microfluidics, has led to the creation of the oviduct-on-a-chip with a U-shaped porous membrane enabling OEC polarization, which can be maintained during long-term culture, therein mimicking tissue and organ-specific micro-architecture (Ferraz *et al*., 2017a, b). It has also been shown that specific tissue morphology and functions are more faithfully mimicked in customized 3D *vs*. 2D systems (Gualtieri *et al*., 2012; Costello *et al*., 2014).

As aforementioned within an *in vivo* context, *in vitro* derived embryos also secrete EVs (Saadeldin *et al*., 2015). These data led us to hypothesize that culture medium supplementation with OEC EVs could initiate a maternal-embryo dialogue beneficial to embryo development. We found that suplementation of *in vitro* embryo culture media with bovine EVs obtained from OECs culture *in vitro* (Lopera-Vásquez *et al*., 2016) and *in vivo* (OF) (Lopera-Vasquez *et al*., 2017) substantially improved *in vitro* produced blastocyst quality.

In addition, the use of OF and UF has been recently used to improve *in vitro* embryo production. One example is a study from our group for which *in vitro* derived embryos were produced and cultured with or without OF and/or UF supplemented media. Low concentrations of OF (days 1 to 4) and UF (days 4 to 8) in serum-free culture indeed supported embryo development and improved embryo quality with OF incorporation resulted in more physiological embryo methylation patterns, whereas UF is thought to have played an antioxidant role (Hamdi *et al.*, 2017).

In conclusion, the oviduct is an important, unique, and interesting secretory organ gaining greater attention owing to increased awareness of embryo- induced changes affecting later stages of development. Answers to fundamental questions foreseeably reside in merging data obtained from advanced complementary *in vivo* and *in vitro* methodologies, all geared at understanding important events of early embryo- maternal communication.
